# Novel Presentation of Pulmonary Atypical Carcinoid Tumor as Acute Pancreatitis

**DOI:** 10.7759/cureus.11063

**Published:** 2020-10-20

**Authors:** Anchit Bharat, Francesca Duncan, Mark Williams

**Affiliations:** 1 Internal Medicine, Indiana University Health Ball Memorial Hospital, Muncie, USA; 2 Pulmonary and Critical Care Medicine, Indiana University School of Medicine, Indianapolis, USA

**Keywords:** atypical carcinoid, acute pancreatitis, neuroendocrine tumors

## Abstract

Pulmonary neuroendocrine tumors (NETs) are a group of rare tumors that pose a high financial burden on patients and the United States healthcare system. The usual presenting symptoms include cough or wheezing, hemoptysis, or chest pain. Due to bronchial obstruction, patients may also present with recurrent pneumonia. Acute pancreatitis has yet to be documented as the initial manifestation of this disease. Atypical carcinoids - a subtype of NETs - are heterogeneous regarding their site of origin, biological behavior, and malignant potential. Studies show that the most common primary tumor site varies by race, with the lung being the most common in white patients and the rectum being the most common in Asian/Pacific Islander, American Indian/Alaskan Native, and African American patients. Certain carcinoid tumors, such as those of the rectum, are over-represented among the Black and Asian populations within the United States, suggesting the role of genetics in the development of this intriguing disease. Furthermore, the pancreas is not a usual site of metastasis for primary lung NET. Our case study describes the rare occurrence of a primary pulmonary NET (atypical carcinoid) metastasizing to the pancreas and presenting as acute pancreatitis.

## Introduction

Pulmonary neuroendocrine tumors (NETs) are a group of rare pulmonary neoplasms characterized by neuroendocrine differentiation [1]. According to the World Health Organization (WHO), they are classified into four types: typical carcinoid, atypical carcinoid (AC), large cell neuroendocrine carcinoma, and small cell neuroendocrine carcinoma [2]. Based on the Surveillance, Epidemiology, and End Results program (2017), the incidence of lung NETs is 1.49 per 100,000 [3-4]. Depending on the pharmacological regimen, the total annual cost for a lung NET patient ranges from $98,713 to $124,383 [5]. This is in contrast to an average of $39,891 for other lung cancers [6]. Not to mention the substantial indirect costs involved with this diagnosis. Hence, it becomes paramount for medical professionals to understand this disease process and be familiar with its rare presentations to provide high-value care. To date, there has been no reported association between lung AC and acute pancreatitis (AP). Our case study will describe this novel presentation and explore possible mechanisms of developing AP in a patient with lung AC.

## Case presentation

**Figure 1:**
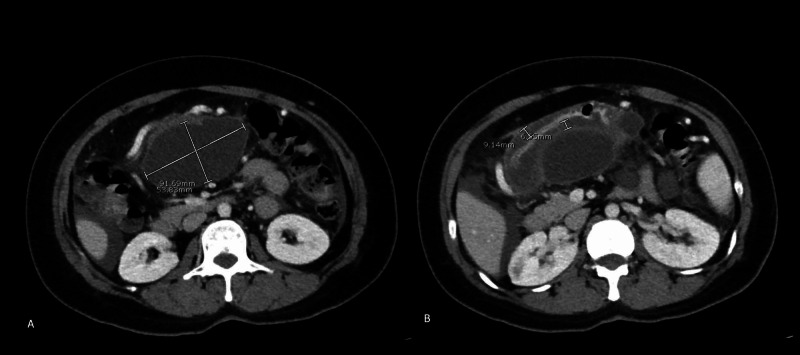
(A): Initial CTAP showing a large dominant fluid collection anterior to the pancreas, extending along the posterior aspect of the mid and distal stomach, arising from the neck of the pancreas, measuring 9.2 x 5.4 cm, with an estimated volume of 250 ml (marked with white pointers); (B): Peripancreatic edema with inflammatory stranding in the pancreatic tail (marked with white pointers) CTAP: computed tomography of the abdomen and pelvis; cm: centimeter; ml: milliliter

A 37-year-old Hakha Chin speaking female without any significant past medical history presented with epigastric pain, nausea, and vomiting for three weeks. She was admitted with acute pancreatitis in the setting of elevated lipase (1153 units/liter) and computed tomography abdomen and pelvis (CTAP) consistent with a large fluid collection arising from the neck of the pancreas (Figures 1A-1B).

**Figure 2:**
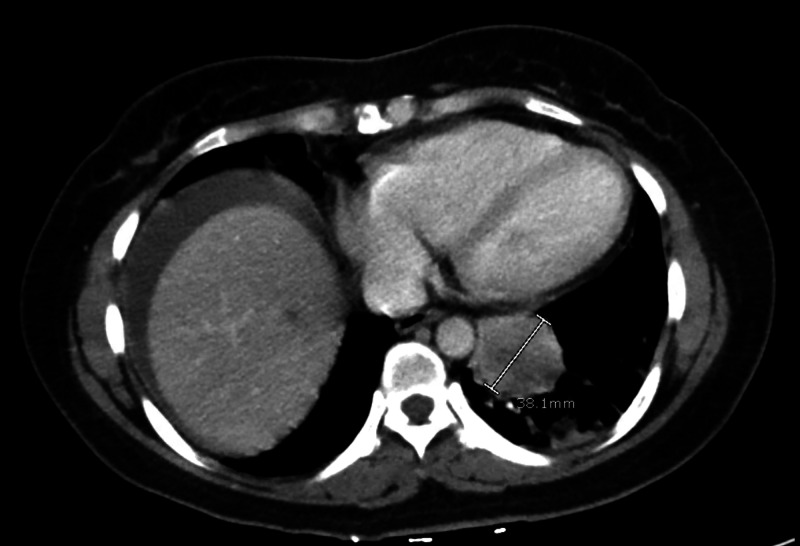
Initial CTAP showing LLL mass-like consolidation measuring 4.1 x 3 cm CTAP: computed tomography of the abdomen and pelvis; LLL: left lower lung; cm: centimeter

**Figure 3:**
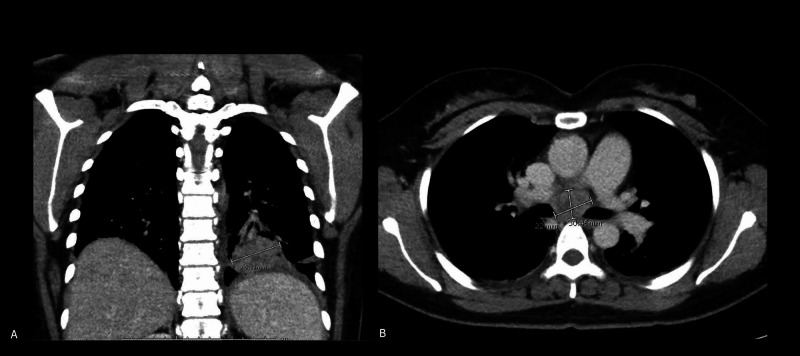
(A): CT chest coronal section showing a 4.0 X 4.1 X 3.0 cm LLL mass with (B): a subcarinal LN measuring 2.7 x 3.1 x 2.9 cm on the transverse section CT: computed tomography; LLL: left lower lung; LN: lymph node; cm: centimeter

Incidentally, CTAP also showed a left lower lung (LLL) mass-like consolidation (Figure 2), which was further evaluated with a CT chest. Imaging demonstrated the LLL mass (Figure 3A), a subcarinal lymph node (LN) (Figure 3B), and multiple suspicious nodular densities in the left breast.

**Figure 4:**
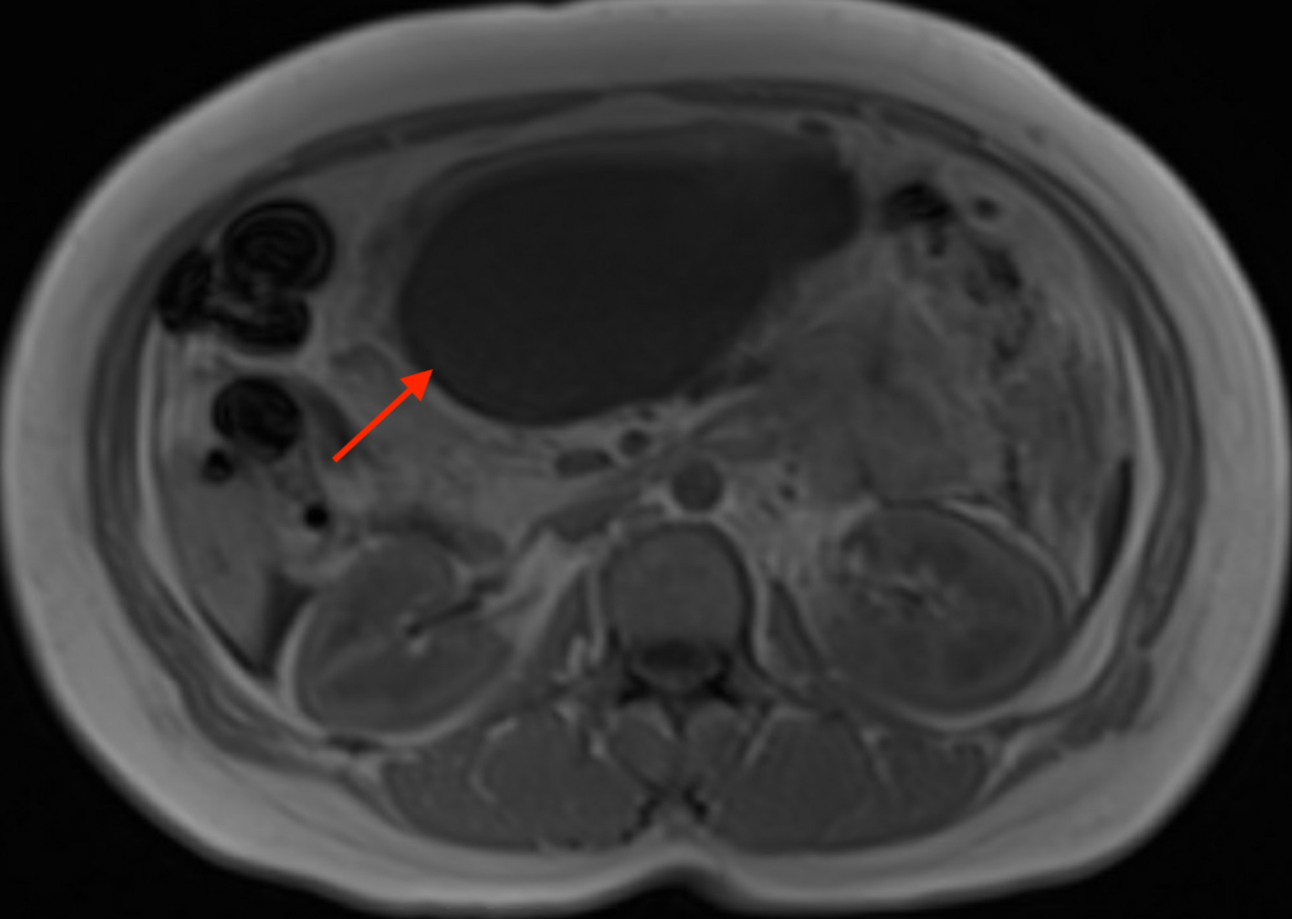
MRCP-confirmed pancreatitis with a rapidly enlarging loculated fluid collection in the lesser sac measuring 11.8 x 10.4 x 6.2 cm (red arrows). Pancreatic ductal dilation noted upstream of the mass MRCP: magnetic resonance cholangiopancreatography; cm: centimeter

**Figure 5:**
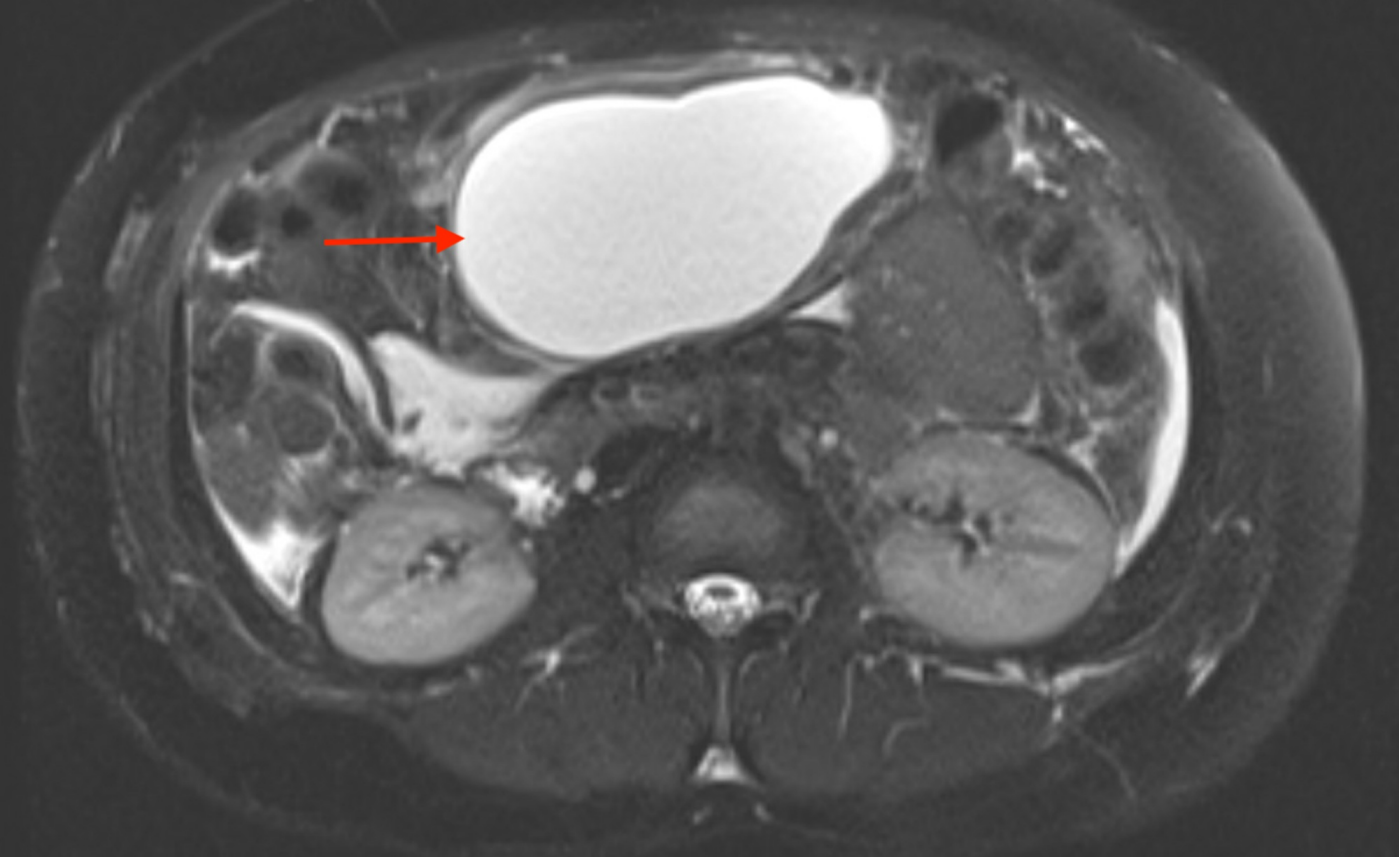
MRCP-confirmed pancreatitis with a rapidly enlarging loculated fluid collection in the lesser sac measuring 11.8 x 10.4 x 6.2 cm (red arrows). Pancreatic ductal dilation noted upstream of the mass MRCP: magnetic resonance cholangiopancreatography; cm: centimeter

The patient was started on bowel rest, aggressive intravenous hydration, analgesics, and anti-emetics. Given a lack of risk factors (alcohol/illicit or over-the-counter drug use, hypertriglyceridemia, hypercalcemia, and family history for hepatic, biliary, or pancreatic disorders) following initial stabilization, a robust workup was undertaken to explore possible etiologies for patients' pancreatitis. Magnetic resonance cholangiopancreatography (MRCP) confirmed the above CTAP findings but was negative for biliary pathologies (Figures 4-5).

**Figure 6:**
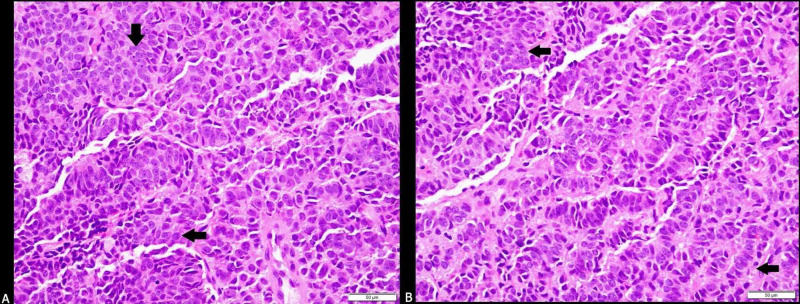
(A&B) Ultrasound-guided subcarinal LN biopsy from mediastinal LN stations 7 and 11 revealing sheets and clusters of neuroendocrine cells with focal pseudo-acinar formation (black arrows) LN: lymph node

An endobronchial ultrasound-guided biopsy from mediastinal LN stations 7 and 11 revealed sheets and clusters of neuroendocrine cells with focal pseudo-acinar formation (Figure 6A-6B).

**Figure 7:**
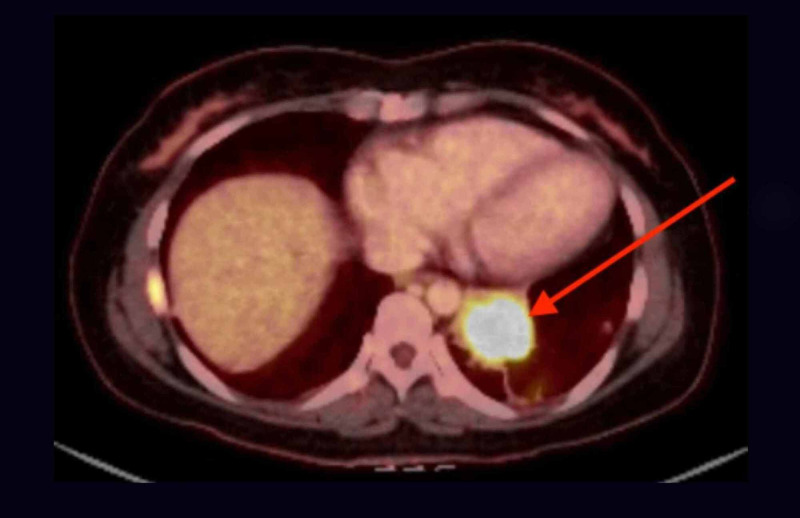
PET/CT showing fluorodeoxyglucose (FDG) avid 4.5 x 4.2 x 3.6 cm LLL mass (red arrow) with max standardized uptake value (SUV) of 12 and FDG avid intramedullary metastasis in the lateral right 7th rib PET/CT: positive emission tomography/computed tomography; FDG: fluorodeoxyglucose; LLL: left lower lung; SUV: standardized uptake value; cm: centimeter

**Figure 8:**
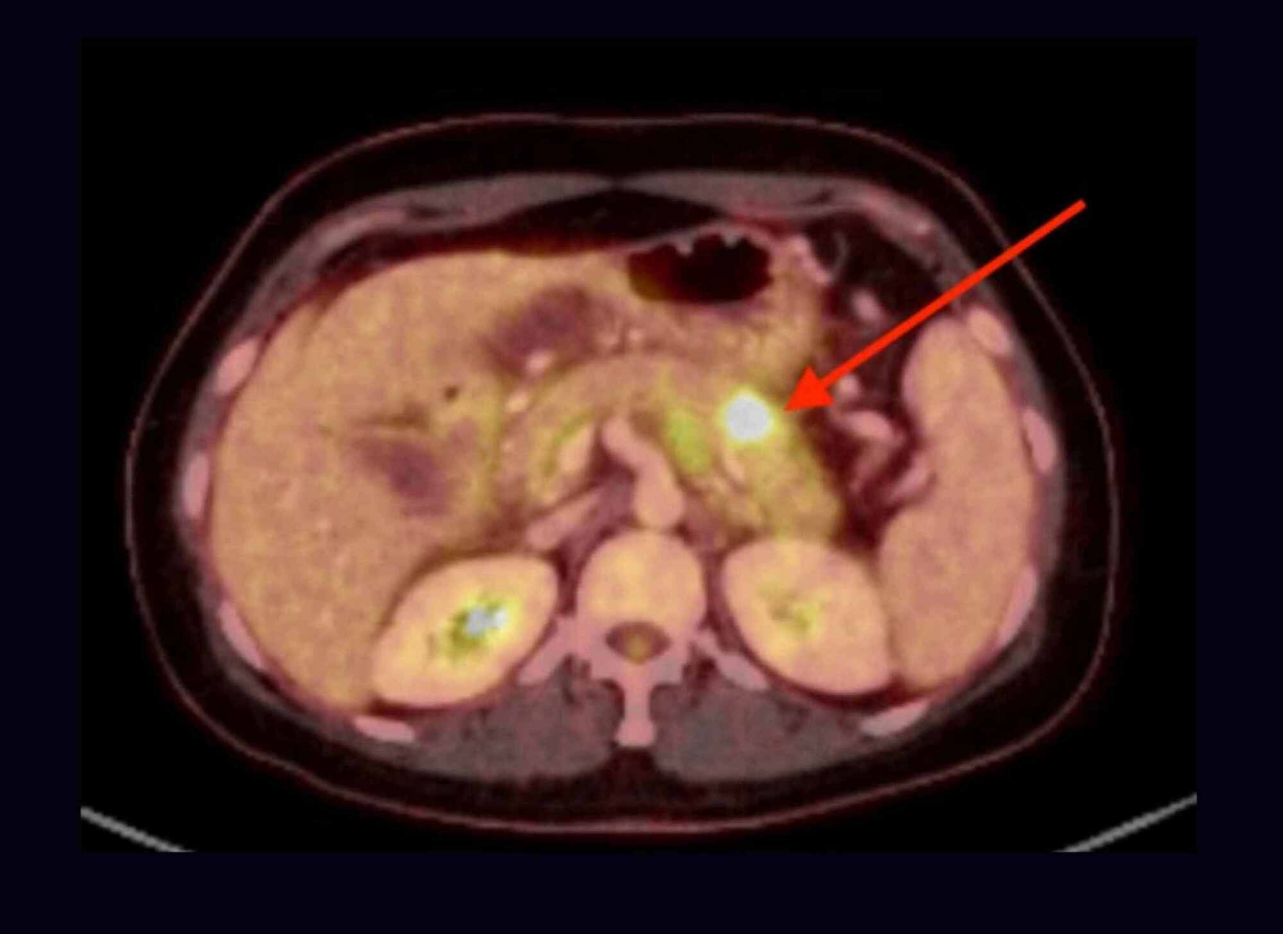
PET/CT showing focal uptake of FDG by a 1.8 cm round and hypodense lesion (red arrow) in the body-tail of the pancreas (max SUV of 11) PET/CT: positive emission tomography/computed tomography; FDG: fluorodeoxyglucose; cm: centimeter; SUV: standardized uptake value

**Figure 9:**
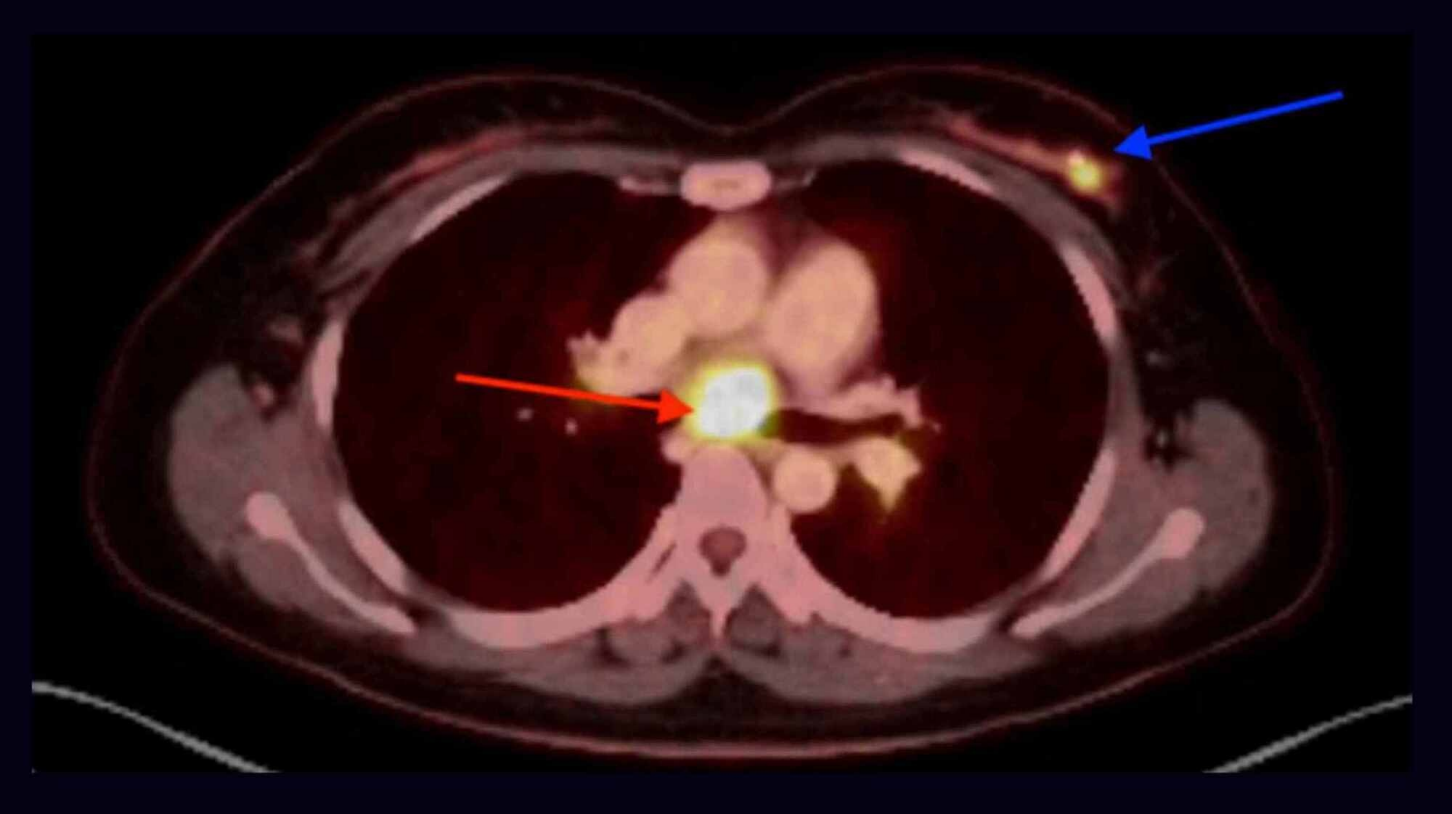
PET/CT showing FDG avid 1.2 x 3.8 x 2.1 cm mediastinal LN station 7 (red arrow) metastatic atypical carcinoid (max SUV of 12.7) and a hypermetabolic left breast mass (blue arrow) with a maximum SUV of 13 PET/CT: positive emission tomography/computed tomography; FDG: fluorodeoxyglucose; cm: centimeter; LN: lymph node; SUV: standardized uptake value

**Figure 10:**
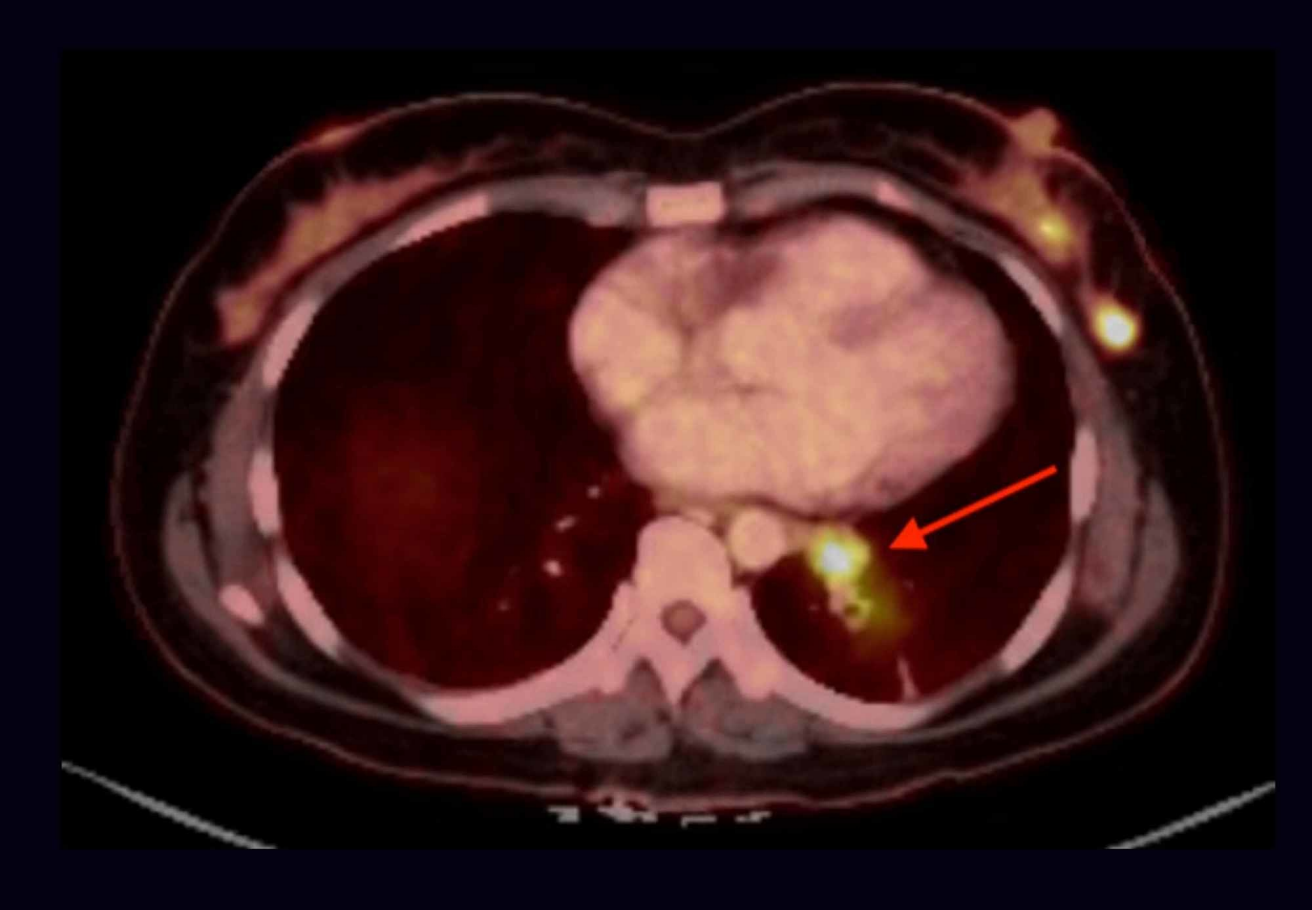
PET/CT showing FDG avid 2.9 x 1.8 x 1.8 cm hilar LN at station 11L-12L (red arrow) metastatic atypical carcinoid (max SUV 12.6) PET/CT: positive emission tomography/computed tomography; FDG: fluorodeoxyglucose; cm: centimeter; LN: lymph node; SUV: standardized uptake value

**Figure 11:**
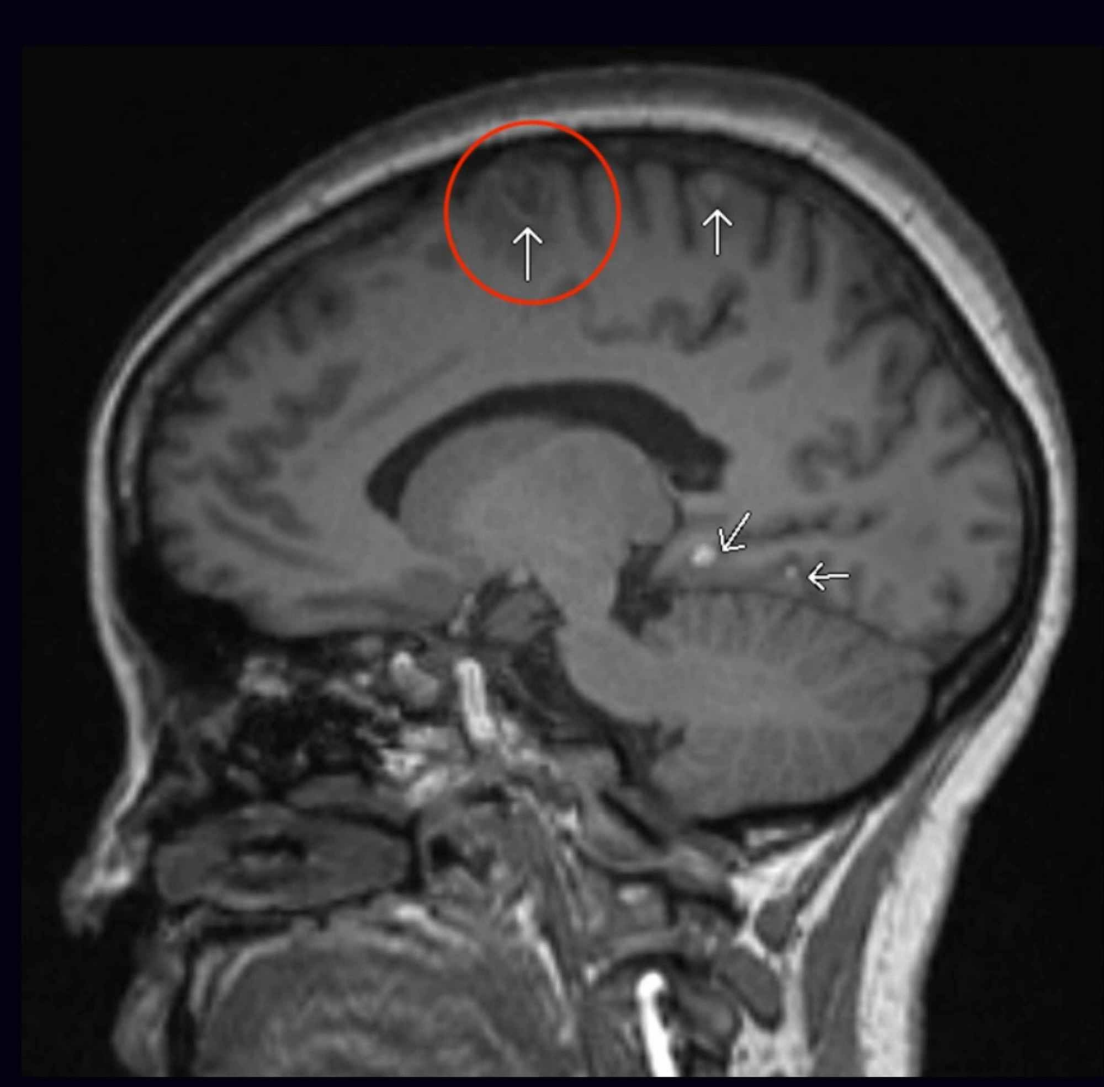
MRI brain sagittal view with numerous contrast-enhancing lesions (white arrows) within cerebral hemisphere measuring between 2-14 mm, the largest lesion being 12 x 13 x 14 mm within the superior left frontal lobe with mild surrounding vasogenic edema (red circle) MRI: magnetic resonance imaging; mm: millimeter

These cells demonstrated strong immunohistochemical staining for chromogranin, synaptophysin, with a Ki-67 labeling index of 5% and were negative for thyroid transcription factor-1, consistent with a metastatic atypical lung carcinoid tumor. Oncology was consulted for treatment recommendations and to establish outpatient care. Following the clinical improvement of pancreatitis, the patient was discharged with a strong outpatient follow-up. Outpatient breast biopsy revealed similar findings as the lymph node biopsy, with tumor cells showing neuroendocrine differentiation and strong immunohistochemical staining for synaptophysin and chromogranin with a Ki-67 < 20%. Positive emission tomography/computed tomography (PET/CT) (Figures 7-10) and magnetic resonance imaging (MRI) brain were obtained for staging (Figure 11).

These studies revealed widespread metastatic disease with approximately 20 cerebral and cerebellar lesions and possible metastasis to the pancreas. In light of Ki-67 being <20%, platinum-based chemotherapy was deemed questionable. The patient was considered a poor surgical candidate and was referred for whole-brain radiation, which she is currently pursuing for palliation.

## Discussion

Due to its origin from proximal airways (as in our patient), lung AC commonly presents with lower respiratory symptoms: cough, wheezing, hemoptysis, chest pain, and recurrent pneumonia in the same pulmonary segment/lobe due to bronchial obstruction [7]. Our patient had no pulmonary symptoms throughout the clinical course even though she had a large LLL mass with significant mediastinal lymphadenopathy in multiple stations. No risk factors were identified for AP through extensive history, physical exam, laboratory workup, and multiple imaging. This presentation remains a medical mystery and the most novel aspect of our case, as AP has not been associated with lung AC before. After discussing with two radiologists, pathologists and gastroenterologists, we hypothesize that the AP was secondary to a metastatic spread of lung AC to the pancreas (Figure 7). This is supported by a focal uptake of FDG by the obstructing pancreatic lesion. The obstruction caused a proximal pancreatic ductal dilation with a failure to visualize the distal duct on MRCP (Figures 4-5), leading to a rapidly enlarging pancreatic cyst with necrotic material seen on imaging and the clinical presentation of pancreatitis. Furthermore, a relatively high SUV, resembling that of the lung AC supports the metastatic theory. So far, there is only one reported case study of such a spread [8]. Pancreatic biopsy remains the gold standard in proving our hypothesis but was deemed unnecessary, as it would contribute little to the management.

Atypical carcinoids are heterogeneous regarding the site of origin, biological behavior, and malignant potential. Genetic and epigenetic alterations may play a seminal role in these factors. Epigenetic studies have shown that the most common primary tumor site varied by race, with the lung being the most common in white patients, and the rectum being the most common in Asian/Pacific Islander, American Indian/Alaskan Native, and African American patients. Certain carcinoid tumors, such as those of the rectum, appear to be over-represented among the Black and Asian populations within the United States, suggesting the role of genetics in the development of this intriguing disease. More work needs to be done to identify potential biomarkers that could impact the diagnostics, prognostic stratification, and planning of personalized therapy [9].

## Conclusions

With this article, we are bringing to light a novel presentation of atypical pulmonary carcinoid, which presented as acute pancreatitis. Racial differences are noted in the primary distribution of this tumor. Appropriate and speedy diagnosis is necessary to provide the patient's timely high-value care.
